# Full color visible imaging with crystalline silicon meta-optics

**DOI:** 10.1038/s41377-025-01888-w

**Published:** 2025-06-18

**Authors:** Johannes E. Fröch, Luocheng Huang, Zhihao Zhou, Virat Tara, Zhuoran Fang, Shane Colburn, Alan Zhan, Minho Choi, Arnab Manna, Andrew Tang, Zheyi Han, Karl F. Böhringer, Arka Majumdar

**Affiliations:** 1 Department of Physics, University of Washington, Seattle, WA 98195 USA; 2 Department of Electrical and Computer Engineering, University of Washington, Seattle, WA 98195 USA; 3 Tunoptix, Seattle, WA 98195 USA; 4 Department of Bioengineering, University of Washington, Seattle, WA 98195 USA; 5 Institute for Nano-Engineered Systems, University of Washington, Seattle, WA 98195 USA

**Keywords:** Applied optics, Metamaterials

## Abstract

Silicon is a common material of choice for semiconductor optics in the infrared spectral range, due to its low cost, well-developed high-volume manufacturing methods, high refractive index, and transparency. It is, however, typically ill-suited for applications in the visible range, due to its large absorption coefficient, especially for green and blue light. Counterintuitively, we demonstrate how ultra-thin crystalline meta-optics enable full-color imaging in the visible range. For this purpose, we employ an inverse design approach, which maximizes the volume under the broadband modulation transfer function of the meta-optics. Beyond that, we demonstrate polarization-multiplexed functionality in the visible. This is particularly important as polarization optics require high index materials, a characteristic often difficult to obtain in the visible.

## Introduction

Silicon is undoubtedly one of the most advanced materials employed in modern-day technologies, forming the backbone for electronic integrated circuits, and sustaining the demands of our technology-driven age. Decade-long development in high-quality and large-scale crystal growth has ultimately enabled this widespread deployment at low cost. Given its ubiquitous usage and fast adaptability for foundry processes, silicon has become of interest in other fields, such as in biomedical applications^1,2^, photonics^3^, and quantum science^4,5^. Particularly for near-infrared photonics (>1200 nm), it emerged as an essential material platform, due to its affordability, high refractive index, well-developed nanofabrication processes and adaptability for foundry processes^6^.

While being well suited for the near-infrared range, it is typically considered incompatible for photonics applications in the visible range due to its large absorption coefficient. However, if the optical path length is extremely short, the loss can be imperceptible and is thus still an attractive material choice. One such class of ultra-thin devices are meta-optics, sub-wavelength structured diffractive surfaces that enable control over the interacting lightfields on the nanometer scale through their localized phase response. Silicon has been used for a range of applications, such as diffractive gratings^7^, monochromatic^8,9^ and polychromatic meta-lenses^10^, resonant nanowires for spectrometer applications^11^, or as structural color elements^12^. Yet, broadband full-color imaging with silicon meta-optics has remained elusive.

Beyond skepticism that crystalline silicon meta-optics can function for the blue spectral range, a major obstacle has been the inherently strong chromatic aberrations that universally impede the functionality of flat optical elements for full-color imaging^13,14^. Particularly, the phase wrapping conditions for the implementation of meta-optics introduce a strong chromatic aberration^15^. Efforts to overcome this obstacle, however, are limited to small numerical apertures or small apertures^16^. Other approaches have been proposed, such as single lenses^17^ or lens arrays^18–20^ in tandem with computational imaging. This can reduce the chromatic aberration in imaging and produce high-quality images using otherwise limited meta-optics.

Herein, we demonstrate crystalline silicon-on-sapphire meta-optics for full-color imaging in the visible and polarization control for multiplexed operation with a single 4 mm aperture. Using commercially available, high-quality silicon on sapphire (SOS) wafers, we achieve full-color imaging with a single meta-optic, with almost negligible loss. We use an inverse design approach to optimize the modular transfer function (MTF) of the meta-optic for a broad wavelength range, considering the material-specific optical constants^21,22^. In essence, this work demonstrates how, contrary to common belief, and other previous efforts, a single layer crystalline silicon meta-optic can in fact be used for full-color imaging in visible.

## Results

**Fig. 1:**
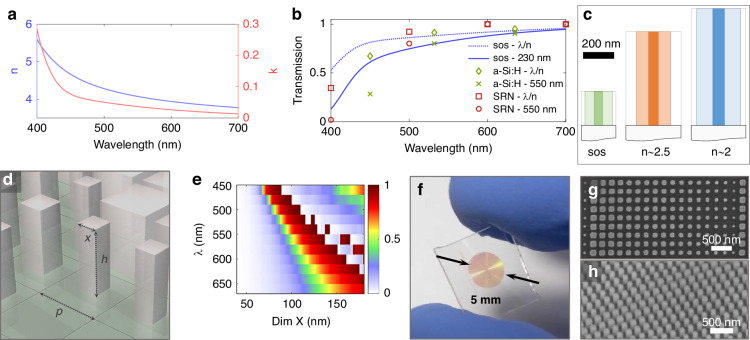
Silicon on sapphire (SOS) visible meta-optics. **a** Refractive index and absorption coefficient for crystalline silicon on sapphire for 400–700 nm. **b** Absolute transmission for SOS compared to a-Si: H and SRN, with values taken from references. **c** Illustrative comparison of the required pillar height to obtain scatterer with a 2*π* phase coverage. Here are pillars compared in SOS, with generic pillars with a refractive index of ~2. 5 and 2. **d** Schematic of nanoscale SOS scatterer with square footprint, defined by period *p*, height *h*, and side length *x*. **d** Phase delay of square scatterer for wavelengths across the visible range, as a function of the side length *x*. The colorbar represents the phase delay, in units of 2*π*. **e** Picture of a sos meta-optic with aperture size of 5 mm. **f** Top down SEM image of a section for a meta-optic. **g** Top-down SEM image of a section of scatterer, highlighting the size control on the nanometer scale. **h** Angled SEM image of the nanoscale SOS scatterer

Several materials have been explored for meta-optics, including SiN^23^, SiC,^24^, HfO_2_^25^, TiO_2_^26^, Nb_2_O_5_^27^, diamond^28^, GaP^18^, polymers^29–31^, and hybrid materials^31^. Arguably, no ideal platform has been identified in the visible wavelength range as each comes with their own challenges and may be suitable for specific aspects. Most materials with transparency below blue wavelengths have a low refractive index, which not only affects the diffraction efficiency^32^ but also hinders multi-functional operation, including polarization control^33^. Moreover, for practical deployment of any meta-optics, the surface needs to be laminated to protect the nano-structures. Lamination materials typically have an index of ~1.5, further reducing the contrast with the low-index materials. Additionally, to the best of our knowledge, most advanced manufacturing foundries are currently working with silicon. This comes from the dichotomy between two different classes of manufacturing facilities: while glass manufacturers can make thick films of oxides, they generally do not have access to high-resolution lithography. On the other hand, semiconductor manufacturers can fabricate high-resolution structures but generally work with thin films. Thus, the ability to fabricate visible meta-optics in silicon can have significant implications for manufacturing. As such, some recent works have investigated structurally disordered silicon (a-Si: H)^34^ and silicon-rich nitride (SRN)^35^ for meta-optics in the visible. However, here we argue that crystalline silicon grown on sapphire (SOS), a material that is already commercially available, is in fact superior for operation over the broad visible spectral range. The refractive index and extinction coefficient of crystalline silicon for the visible wavelength range are plotted in Fig. 1a. At 400 nm, we obtain an extinction coefficient *k* of 0.28, which drops to ~0.08 at 450 nm and further reduces to 0.05 at 500 nm and ~0.02 at 650 nm. At the same time, the refractive index has a value of 4.7 at 450 nm, and to achieve a full 2*π* phase shift, a film of ~100 nm thickness would suffice. In that case, even for a full film, roughly 80% of the light would be transmitted, and once nanostructured, the effective absorption would decrease further.

For a quantitative comparison, we consider the absolute transmission through an unstructured film in two cases, namely one of thickness of *λ*/*n* (suitable for monochrome operation) and for a thickness that would enable full 2*π* phase coverage at 650 nm (suitable for visible broadband operation), corresponding to ~230 nm for SOS, and ~550 nm for a-Si: H and SRN. We consider the significantly higher refractive index of silicon relative to other materials and thus show a better comparison than only considering the extinction coefficient or the ratio of *n*/*k*. The resultant transmission values are plotted in Fig. 1b. Notably, within the blue spectral range SOS demonstrates significantly higher transmission than the other materials in both scenarios, while having on par transmission within the green and red spectral range. This shows that the refractive index for a-Si: H and SRN reduces more significantly than the extinction coefficient *k*, and thus as a net effect the total transmission is lower, if we consider thin films of thickness suitable to achieve the same phase coverage, in particular for the blue spectral range. There are variations in measurements of *n* and *k* for silicon in the literature, and we have discussed this point further in the Supplementary Information. In addition, we note that other high-index materials such as GaP, yield a smaller refractive index (~3.2–3.8) than silicon on sapphire while maintaining a similar extinction coefficient (~0.075 at 450 nm) as further illustrated in the Supplementary Information^18,36,37^.

To emphasize the size reduction attainable thanks to the high refractive index of silicon, we illustrate the required pillar heights in Fig. 1c to obtain a phase shift of 2*π* at ~650 nm, in comparison with materials with a refractive index of ~2.5 (e.g., TiO_2_) or ~2 (e.g., SiN). As shown in this work, for computationally assisted broadband operation, an aspect ratio of ~4 suffices to obtain a desirable phase coverage over the broadband visible range. In comparison, materials with a refractive index of *n* = 2. 5 would require a height of ~ 600 nm^38^, and for *n*~2, requires 750 nm, with aspect ratios on the order of ~10 or larger to obtain a phase coverage of at least 2*π*^39^. The capability to obtain smaller aspect ratios is inherently more robust to fabrication errors, such as severe undercutting or uneven etch depth in small gaps, which may occur during dry etching^40^. This gives this platform a clear advantage, requiring less arduous optimization and better transfer of etch recipes from one design to another. Moreover, a smaller refractive index requires a larger periodicity to effectively achieve the desired phase coverage. However, a larger period decreases the overall efficiency as the phase is not as effectively sampled with a larger period or can even lead to aliasing effects^41–43^. Also, in previous works clear advantages of higher refractive index has been shown, especially for higher NA^13,33,44^.

For the SOS meta-optics designs, we assume pillars with a square footprint, schematically illustrated in Fig. 1d, whereas their phase delay is determined by the period, height, and side length of these pillars. In this work, we consider a height of 230 nm, which is found to be at the right trade-off to provide sufficient phase coverage in the visible range, while at the same time minimizing the loss at shorter wavelengths. The resultant phase coverage in the visible range for this pillar is shown in Fig. 1e.

We used a standard nanofabrication approach, using electron-beam lithography (EBL) to write the meta-optic pattern in a resist, followed by hard mask evaporation (AlO_x_), and reactive ion etching (RIE). This allowed us to fabricate apertures up to 5 mm diameter as shown in Fig. 1f. Specific intermediate steps and conditions are detailed in the “Methods” section. Given the shallow etch height and the fact that silicon is one of the most well-developed materials with well-defined etch conditions, a high degree of control over the structural integrity is easily achievable down to the nanometer scale. This fact is highlighted in several scanning electron microscope (SEM) images in Fig. 1g, h. Noticeably, the relatively low aspect ratio facilitates high accuracy over the fabrication, while maintaining smooth pillar sidewalls. In fabrication we observe negligible deviation from the intended designs, summarized in the Supplementary Information.

As color information is crucial in many imaging scenarios, broadband operation over the entire visible range is often critical. Meta-optics, however, are known to have severe chromaticity, typically exhibiting an inverse proportionality of the focal length with wavelength for a fixed metalens (hyperboloid phase mask). This has been mitigated in previous works by either using dispersion engineering^16,45–47^ or an end-to-end^17^ design approach, where the broadband sensor performance is considered in tandem with a computational back end. However, the former is typically limited to small aperture sizes or a small NA, which may preclude their integration with functional devices. Herein, we use a recently developed inverse design approach, named MTF-engineering^21,22^, where we optimize the broadband volume of the MTF of the flat optic. This optimization also directly considers the strongly varying optical constants of the material throughout the visible range. We assume a rotationally symmetric phase profile for optimization of the phase and amplitude response of scatterers for wavelengths in a range of 460–700 nm (with step size of 20 nm). We emphasize here that this design in comparison to polychromatic RGB meta-optics^10,48–50^, does not only cover 3 specific channels (R, G, B) but operates over the entire visible wavelength range as further demonstrated in the Supplementary Information. With this design approach, we designed two f/2 aperture meta-optics with diameters of 0.5 mm and 1 mm. We further considered a meta-optic (5 mm diameter) with an extended depth of focus design approach, adopted from a prior work^50^, which is detailed in the Methods section. To obtain a baseline comparison of the meta-optic functionality, we further fabricated hyperboloid metalenses (detailed in the Supplementary Information) with 0.5 mm apertures, designed for specific wavelengths of 450 nm (*B*), 550 nm (*G*), and 650 nm (*R*).

**Fig. 2:**
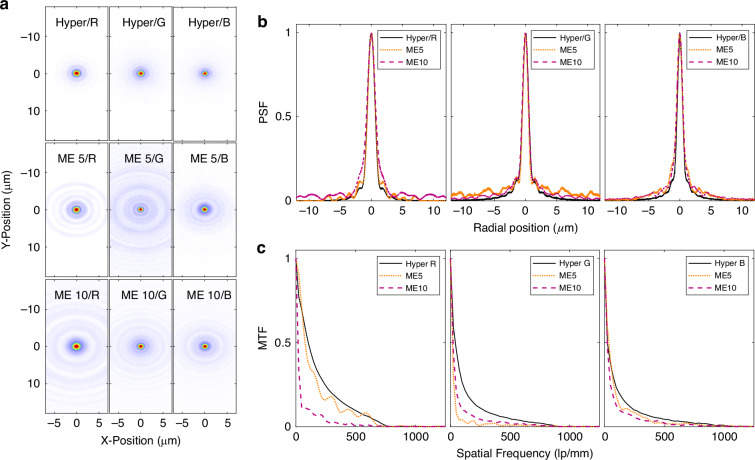
Optical characterization. **a** PSF measurements of SOS meta-optics. The top row is measurements of individual hyperboloids, specifically designed for wavelengths of 450 nm, 550 nm, and 650 nm. Middle row: PSF measurements for an MTF engineered meta-optic with 500 µm aperture. Bottom row: PSF measurements for an MTF engineered meta-optic with 1 mm aperture. **b** Cross sections of the PSF in direct comparison. **c** The MTF derived from the PSF measurements. Hyper refer to individual metalenses with hyperboloid phase profiles, optimized for a single wavelength. These are compared to single-aperture meta-optic ME5 (500 µm aperture) and ME10 (1 mm aperture)

We characterized the basic properties of all lenses by measuring their PSF, as summarized in Fig. 2. Collimated light from a light emitting diode (LED) with a spectral width at half maximum of about ~15 nm was transmitted through a meta-optic and then collected using a microscope setup with a 50X, 0. 75 NA objective. The NA of the objective is sufficiently large to facilitate full capture of light that forms the PSF. The respective measurements of the hyperboloid metalenses are shown in comparison to the MTF-engineered meta-optic in Fig. 2a, whereas their cross sections are compared in Fig. 2b.

Notably, the hyperboloid metalenses are designed for different colors, and while these clearly form the most tightly confined PSF in all three cases, we emphasize that these are three separate apertures, which are individually not suitable for capturing the other wavelength ranges. In contrast, the MTF engineered meta-optics are capable of closely capturing all wavelengths simultaneously although more power is contained in the side lobes of the PSF. Importantly, these inverse designed meta-optics work at higher NA and larger aperture than previously employed dispersion-engineered meta-optics, which are typically limited to only a few Fresnel zones. To benchmark this work to others in the field, we summarized several meta-optic full color imaging works, with respect to aperture size, bandwidth, and Fresnel number as described in the Supplementary Information. Our work is at the intersection of achieving low aspect ratios with small heights, enabling a robust and cost-effective fabrication approach, while at the same time we also achieve larger Fresnel numbers, which indicates the capability to of achieving higher focusing power with larger apertures.

Due to the stronger sidelobe, the resolving capability of the broadband meta-optic is, however, limited. To analyze their limitations, we have derived the MTF from the PSF measurements as shown in Fig. 2c. We can see that the hyperboloid metalens achieves the highest volume under the MTF curve. However, these are only for a single wavelength. In comparison the single aperture MTF-engineered meta-optic shows curves relatively close to those of the three individual hyperboloid metalens. This highlights the validity of the design approach to achieve broadband meta-optics. We further emphasize that, while the contrast is lower in this measurement, a computational backend can enhance the imaging performance as further demonstrated below.

**Fig. 3:**
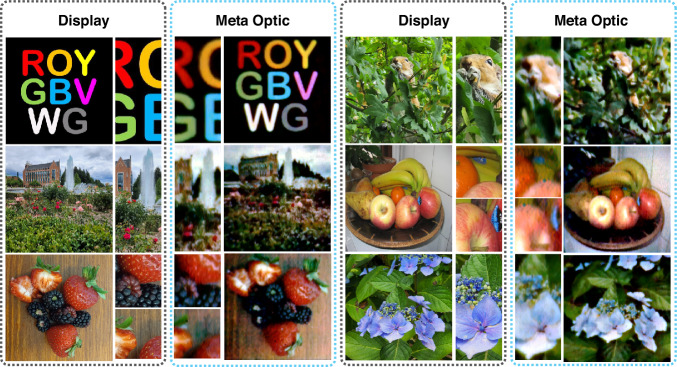
Full color imaging capability. Examples of full color imaging are collated in this figure, comparing images displayed on an OLED display and the image after computational reconstruction using a 1 mm MTF-engineered meta-optic

To demonstrate full-color imaging using the SOS MTF engineered meta-optic, images were projected on an organic light emitting diode (OLED) screen and then imaged via the meta-optic placed ~50 cm in front. A microscope setup captured the image produced by the meta-optic. For a clear assessment, we used images with various color contrasts in red, green, and blue, as well as with different features. The results are summarized in Fig. 3, comparing images projected on an OLED screen against images captured with the MTF-engineered meta-optic and computational reconstruction. For the computational reconstruction we used Wiener deconvolution, which is fully scene independent and only relies on the beforehand measured PSF of the meta-optic. In addition to reduce the noise after deconvolution, we used a BM3D block-filtering algorithm, which is a patchwise noise filtering approach^51,52^.

Notably, we observe that the meta-optic is capable of full color imaging, evidenced by capturing features with red, green, and blue colors, while preserving color contrasts and image details. This is made possible by the inverse design approach, which is detailed in the Supplementary Information, combined with the computational imaging. For instance, in the image with colored letters, one can clearly identify individual colors, with a crispness of the image close to the displayed image. Even smaller details, such as individual seeds of a strawberry, or the individual buds of a blue flower can be identified, as seen in the magnified images. Only for extremely fine image details the meta-optic is not capable of capturing details, such as the letters on the sticker on the apple.

We have also conducted comparable imaging experiments with SiN meta-optics of the same parameters, which show on-par performance of SoS with SiN meta-optics, presented in the Supplementary Information.

We note that some variations in the colors between the original and reconstructed image are likely due to discrepancies in the white balancing during image capture and could be further adjusted to improve the likeness of the captured images with the original. However, this can be considered only a fine adjustment knob and would not further improve the imaging quality, which is ultimately limited by the MTF of the system, measured in Fig. 2. Moreover, it would be possible to apply recently developed neural network backends which would improve the image quality slightly. Yet, the ultimate limit of the achieveable resolution is given by the cut-off frequency for the MTF plots, shown in Fig. 2. Once the MTF contrast falls off to zero, the computational reconstruction is not possible. However, these cut-off frequencies appear at appreciably high values to enable imaging up to a certain detail^50^.

We emphasize here that these images were reconstructed from captures of a single meta-optic aperture, which are capable of imaging throughout the visible wavelength range, thanks to the MTF engineering approach.

**Fig. 4:**
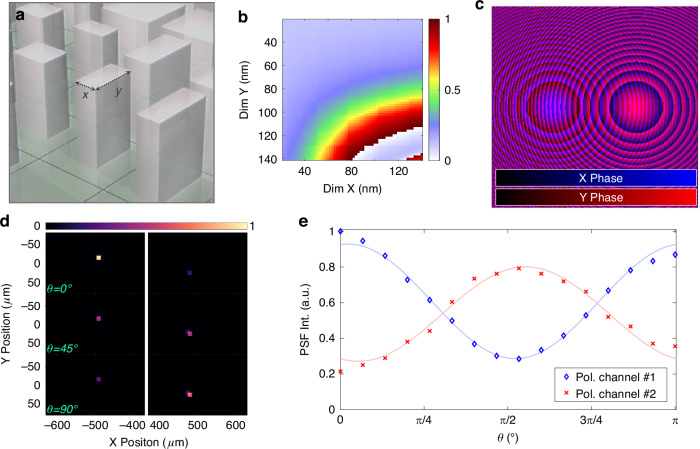
Polarization selective operation of SOS meta-optics. **a** Symmetry-breaking of the scatterer, introducing another degree of freedom, Y. **b** Phase response for scatterer with rectangular footprint at a wavelength of 550 nm for y-polarized light. **c** Illustration of a single aperture meta-optic with two different phase profiles implemented for orthogonal polarizations. The phase mask implemented for *x*-polarized light is shown on a blue colorscale. The phase mask implemented for *y*-polarized light is shown on a red colorscale. **d** Direct measurement of the focal spots on a sensor for polarizations correspond to 0°, 45°, and 90°. **e** Contrast in the two focal spots of the polarization multiplexed meta-optic

To further highlight the capability of the SOS platform for applications in the visible spectral range, we fabricated polarization multiplexing metalenses in the green wavelength range. Leveraging the high refractive index of silicon provides a strong advantage over other materials with lower index, which makes SOS particularly suitable for this application. For this purpose, we break the symmetry of the individual square scatterer as schematically shown in Fig. 4a. By introducing a variable side length Y, we can engineer the phase response in two orthogonal directions independently. The phase delay, as function of the X and Y dimensions for *y*-polarized light using such scatterer, is illustrated in Fig. 4b. We note that the phase delay for the orthogonal polarization corresponds to the transposed matrix. The available scatterer variety facilitates the implementation of overlapping independent phase profiles. Specifically, we demonstrate this capability here by encoding two hyperboloid metalens profiles with a focal spot offset, for *x* and *y* polarization, as illustrated in Fig. 4c. For this purpose, we demonstrate a 2 mm aperture and 4 mm focal length, and 500 µm offset of the focal spot.

In experiment, we illuminated the meta-optic with a polarized laser at a wavelength of 550 nm and measured the PSF directly on a sensor. By additionally varying a half-wave plate in the optical path, we observe that under rotation the two focal spots of the single aperture meta-optic vary in intensity, as shown in Fig. 4d. The relative change in intensity upon rotation of the half wave plate is summarized in Fig. 4e, fit with an intensity according to Malus’ law.

## Discussion

Contrary to previous works, we have shown how commercially available crystalline silicon, grown on sapphire, is suitable for full-color imaging, and in fact outperforms other high-index material platforms in terms of its loss. Using an inverse design approach, we could consider the strongly varying nature of the optical constants in the visible range, while optimizing meta-optics for full-color imaging. Specifically, we optimized f/2 meta-optics up to a 1 mm diameter to support broadband imaging behavior using the volume under the MTF curve as the figure of merit. In combination with a simple computational backend, this allowed us to achieve high-quality imaging over the full visible spectral range. We further demonstrate multiplexed focus control by encoding polarization-dependent phase profiles through symmetry-broken scatterer. We highlight that our work is at the intersection of achieving low aspect ratios with small heights, which is favorable for a robust and cost-effective fabrication approach, while at the same time we also achieve larger Fresnel numbers, which indicates the capability of maintaining higher focusing power with larger apertures.

In essence, this work demonstrates the extension of silicon meta-optics from the IR range to the visible and an inverse design approach to overcome the strongly changing conditions of the optical constants. This work enables large scale silicon meta-optics for full color imaging systems and beyond that unlocks further capabilities in future directions, such as for various forms of sensing^53–56^, object classification^57–59^, spectrometer applications^60–63^, and a myriad of other future directions where meta-optics hold the potential to play a key role^63^.

## Materials and methods

### Fabrication

We purchased silicon on sapphire wafers, with a film thickness of 230 nm from University Wafer. The wafer was diced into square chips using a Disco Saw Wafer dicer, then briefly cleaned in an ultrasonicating bath of Acetone and subsequently IPA. Chips were then dry-cleaned using a barrel etch step (O_2_, 100 W, 15 s).

For nanolithography, we then applied a positive resist (ZEP 520-A) onto the sample (~400 nm), followed by baking on a hot plate at 180 °C for 3 min. Subsequently, a conductive polymer layer was applied on top of the ZEP layer (DisCharge H_2_O). The design was then patterned into the resist using a 100 keV electron beam (JEOL JBX6300FS) at a dose of ~275 µC cm^−2^. After EBL, we first dissolved the conductive polymer layer in a short IPA bath and subsequently developed the resist in a beaker with Amyl Acetate for 2 min at room temperature. In the next step, we descummed the patterned regions of the sample in a short barrel etch step (O_2_, 100 W, 15 s). We then deposited a layer of ~50 nm Al_2_O_3_ using electron beam evaporation. The resist was then lifted off overnight in an NMP bath at ~100 °C on a hot plate. After cleaning in solvents and a further descum step, the SOS was etched using a mixture of C4F8/SF6 in an inductively coupled reactive ion etcher (Oxford PlasmaLab System 100). After etching, the sample was cleaned in solvents (Acetone, IPA), and we subsequently fabricated metal apertures to eliminate spurious light during measurements. For this purpose, 3 µm thick negative photoresist (NR9G-3000PY) was spin-coated onto the sample followed by 2 min baking on a hotplate at 110 °C. The coated sample was then exposed using a photolithography system (Heidelberg DWL66+) followed by 90 s post exposure bake at 110 °C. The photoresist was then developed for 1 min in AD-10. Two hundred nanometers chromium was then deposited using e-beam evaporation (CHA Solution). After that lift off was performed by submerging the sample in acetone for 5 min followed by 2 min IPA rinse.

### Measurements

We measured the refractive index of the SOS using an ellipsometer (Woollam RC2). To measure the PSFs of the optics at various wavelengths we transmitted light of a fiber-coupled LED with a wavelength of either 455 nm, 520 nm, or 625 nm through the meta-optic. The PSF was then collected in a custom-built microscope setup, consisting of a 50× Objective (0.75 NA), a 200 mm tube lens, and a color camera (ProSilica GT1930C).

For imaging, we placed a self-luminescent OLED screen ~50 cm in front of the meta-optic, while the image produced by the meta-optic was then captured using the same relay system.

Images displayed on the OLED screen were either taken by one of the authors or taken from the INRIA Holiday dataset^64^.

## Supplementary information

Supplementary Information

## Data Availability

Data available on request from the authors.
