# Supplementary material for: Full color visible imaging with crystalline silicon meta-optics
Source: Light Sci Appl. 2025 Jun 18;14:217. doi: 10.1038/s41377-025-01888-w (PMC12177033; doi:10.1038/s41377-025-01888-w)
Supplement: Supplementary file 1 — Supplementary Information [file 41377_2025_1888_MOESM1_ESM.pdf]

Supplementary Information for

Full Color Visible Imaging with Crystalline Silicon Meta-Optics

*Johannes E. Fröch[1, 2, *], Luocheng Huang[2], Zhihao Zhou[2], Virat Tara[2], Zhuoran Fang[2], Shane Colburn[2, 3], Alan Zhan[3], Minho Choi[2], Arnab Manna[1], Andrew Tang[2], Zheyi Han[2], Karl F. Böhringer[2, 4, 5], Arka Majumdar[1, 2, *]*

1: Department of Physics, University of Washington, Seattle, 98195, WA, USA

2: Department of Electrical and Computer Engineering, University of Washington, Seattle, 98195, WA, USA

3: Tunoptix, 4000 Mason Road 300, Fluke Hall, Seattle, WA, 98195 USA

4: Department of Bioengineering, University of Washington, Seattle, WA, 98195, USA

5: Institute for Nano-Engineered Systems, University of Washington, Seattle, WA, 98195, USA

S1. Comparison of ellipsometry measurements of silicon in other works

We note that there are variations of n and k of crystalline silicon in the literature. This likely arises due to differences in material quality and some uncertainties in the fitting of optical constants during ellipsometry measurement. However, to underline our argument that the crystalline silicon platform provides better performance than a-Si:H or SRN in the blue range we further discuss measurements on the optical constants from literature and show that even considering the strongest absorption values reported, it outperforms the other platforms in the blue wavelength range, while on par in the green and red range.

Literature values for n and k (obtained from ref [1] on November 2 2023) are summarized in Figure S1a and b, respectively, and compared to the n and k measurement of our work, as well as values for a-Si:H and SRN from works [2] and [3], respectively. The absolute wavelength dependent transmission was calculated with equation 1 and 2, which are plotted for a thickness of λ/n in Figure S1c, as well as for a thickness of 230 nm for Silicon, and 550 nm for a-Si:H, and SRN, which provide a full ~ 2π phase shift over the broadband range.

We furthermore considered GaP as another material with high refractive index and loss in the visible.[4,5]

We note that authors in [2] refer to [6] for the value of n and k. Although this extinction coefficient is higher than reported in other works, as well as the value obtained in our work, it still shows better performance in terms of transmission in the blue range compared to their respective work.

At the same time crystalline silicon provides on par performance to the other materials discussed. This clearly shows that simply decreasing the absorption coefficient, while not considering the reduction in the actual refractive index does not necessarily improve the performance in terms of transmission of a metasurface.

$$(1)\ \alpha = 4\pi \frac{k}{\lambda}$$

$$(2)\ T = \exp(-\alpha \cdot t)$$

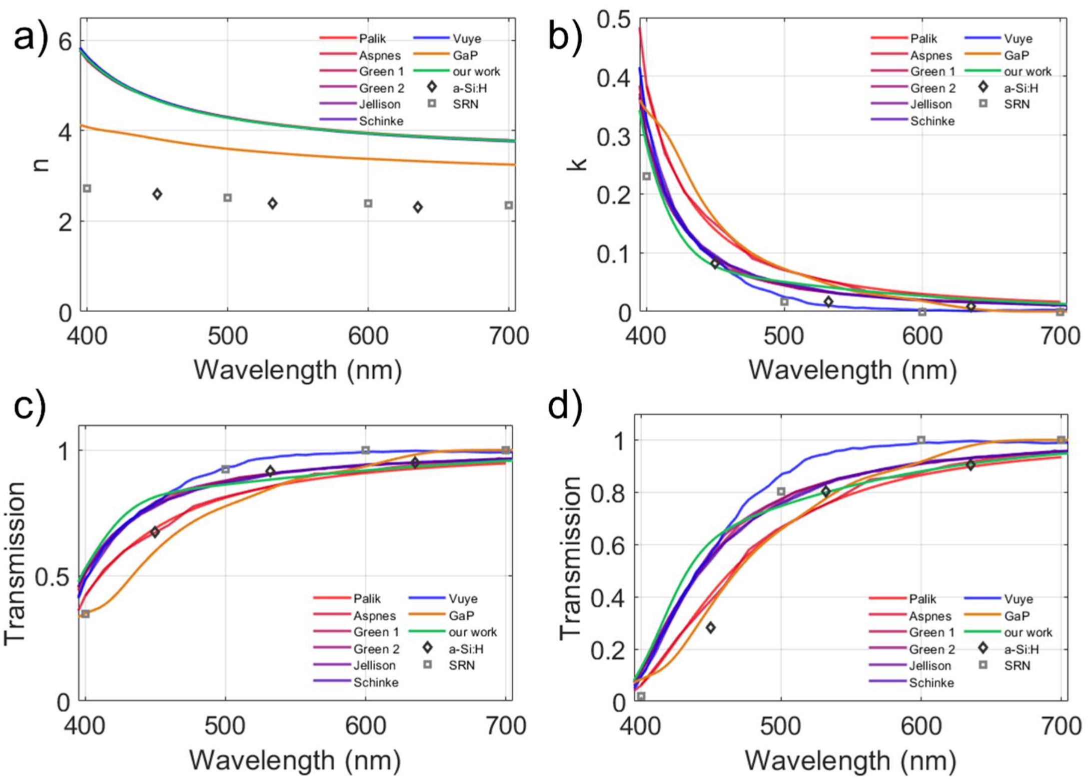

Figure S1. a) Comparison of the real part for the refractive index. b) Comparison of the extinction coefficient of the refractive index. c) Transmission for a film thickness of λ/n. d) Transmission for a film thickness of 230 nm (for Si) or 550 nm (for a-Si:H or SRN). Palik refers to [6], Aspnes refers to [7], Green 1 refers to [8], Green 2 refers to [9], Jellison refers to [10], Schinke refers to [11], Vuye refers to [12], GaP refers to [4], a-Si:H refers to values from [2], SRN refers to values from [3].

S2. MTF engineering design approach

The specific design method has been utilized in Refs [13,14], but for completeness has been reproduced here. For design we employ an automatic differentiation framework. First a deep learning maps the scatterer geometry to wavelength dependent phase. This type of mapping is differentiable, enabling the optimization of scatterers. A plane wave (for different wavelengths (λ)) is transmitted through an initial phase-mask and then propagated to the sensor plane using the band-limited angular spectrum method. From the PSF at the sensor plane, the MTF is calculated as the Fourier transform. The Figure of Merit (FOM) for optimization is defined as given below, whereas the Strehl Ratio (SR) is defined as the ratio of the volume under the MTF curve over the volume under a diffraction limited MTF curve.

$$FOM = -\sum_{\lambda} \log\big(SR(\lambda)\big) = -\log\left(\prod_{\lambda} SR(\lambda)\right)$$

This FOM is minimized when $SR(\lambda)$ is high, when all entries $SR(\lambda)$ are similar, as the geometric mean is highest when all terms are equal. The resultant PSFs after simulation for a 1 mm MTF engineered meta-optic are summarized in Figure S2.

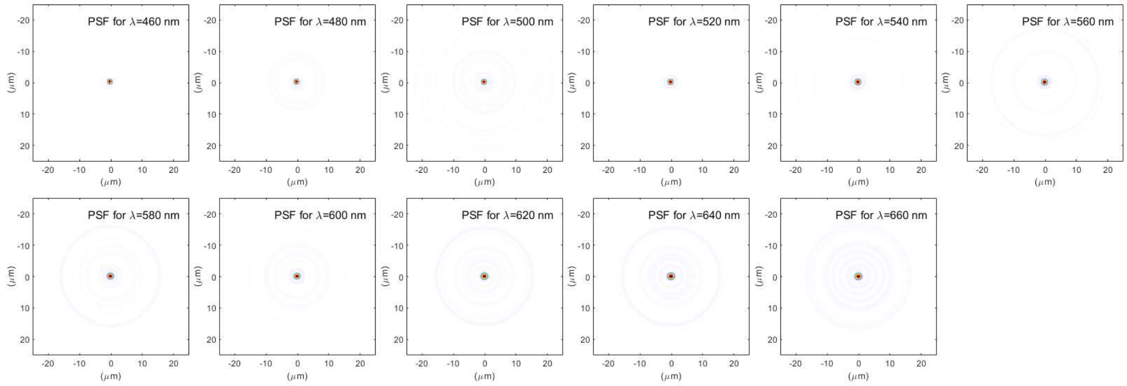

Figure S2. Simulated PSFs for wavelengths across the visible spectrum for a 1mm MTF engineered meta-optic.

S3. Comparison to other meta-optic for full color broadband imaging in the visible range.

To compare this work with others in the field, we provide a comprehensive table summarizing key parameters achieved for full color imaging in the visible across various material platforms and designs. We note that we primarily consider works that utilized designs with point spread functions suitable for the visible wavelength range, and did not consider works that heavily rely on learned reconstruction. Specifically, we compare diameter (d), height (h), aspect ratio (AR), refractive index (n), material, wavelength range, numerical aperture (NA) and Fresnel number (FN), calculated as $FN = \frac{d^2}{4f\lambda}$. Towards useful applications, larger device apertures (> 1 mm) are required to achieve reasonable performance on a sensor with reasonable exposure times. At the same time a smaller height and smaller AR alleviate fabrication challenges. This overall makes large Fresnel numbers necessary in design, and smaller height/AR desirable. As outline in the table, this work achieves both of these properties relative to others, thanks to the properties of the thin crystalline silicon on sapphire.

Table 1. Comparison of meta-optics and multi layer diffractive optical elements for broadband imaging in the visible range. For comparison we include the aperture diameter, height, aspect ratio, refractive index n, the material, wavelength range, numerical aperture (NA), and Fresnel Number (FN).

Year	d (μm)	h (μm)	AR	n	Material	λ (nm)	NA	FN	Ref
**2018**	20	0.6	12	2.5	TiO_2	470 - 670	0.2	4	[15]
**2018**	220	0.6	12	2.5	TiO_2	470 - 670	0.02	4	[15]
**2018**	50	0.8	18	2.5	GaN	400 - 660	0.106	5	[16]
**2018**	370	2.6	5	1.61	Photoresist	450 -750	0.18	56	[17]
**2019**	26.4	0.6	10	2.5	TiO_2	460 - 700	0.2	5	[18]
**2019**	21.6	0.8	18	2.5	GaN	400 - 660	0.216	5	[19]
**2020**	3145	2.6	4	1.61	Photoresist	450 - 1000	0.3	682	[20]
**2021**	20	2.5	5	1.57	IP-DIP	450 - 1700	0.27	3	[21]
**2021**	200	2.5	5	1.57	IP-DIP	450 - 1700	0.04	4	[21]
**2021**	500	0.7	10	2	SiN	400 - 700	0.24	112	[22]
**2022**	10240	1	7.5	1.63	Photoresist	400 - 1100	0.1	686	[23]
**2023**	50	1	20	2.5	TiO_2	400 - 1000	0.164	6	[24]
**2023**	1000	0.7	10	2	SiN	400 - 700	0.24	225	[13]
**2024**	10000	0.8	10	2	SiN	450 - 650	0.24	2248	[25]
**2025**	1000	0.23	4	4	SoS	460 - 660	0.24	221	This Work
**2021**	2000	0.6	12	2.5	TiO_2	488 / 532 / 658	0.7	1842	[26]
**2022**	10000	0.6	12	2.5	TiO_2	488 / 532 / 658	0.3	2956	[27]
**2024**	200	0.3	6	3.3	GaP	460 / 530 / 620	0.77	227	[28]
**2025**	10000	0.7	8	1.5	Photoresist	450 / 532 / 635	0.7	9212	[29]

S4. Fabrication accuracy

To fabricate the crystalline silicon on sapphire meta-optics, we designed square pillars with a side width limited between 50 nm and 175 nm. In Figure S3, an SEM image of a larger area is shown. As can be seen some of the smallest pillars were not patterned accurately. However, this might have negligible influence on the final performance, as these pillars are designed with a phase shift of close to 0. Otherwise, a higher resolution image, overlayed with the outline of the intended design directly shows an extremely close match between the designed structure and the fabricated device. Variations between the pillar foot print and the target size are not measurable at this scale.

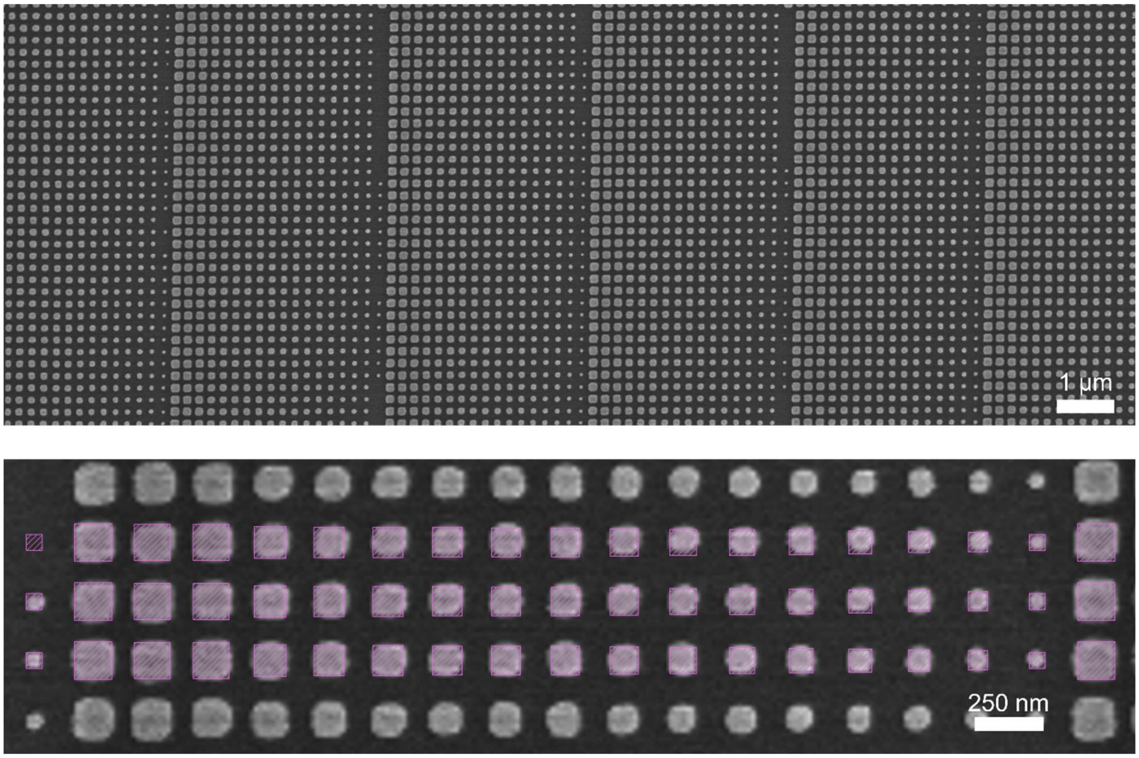

Figure S3. SEM image of a meta-optics section and magnified view overlayed with the intended design (purple).

S5. Optical Setup for PSF measurement

To accurately measure the PSF of the various meta-optics we utilized a microscope setup as shown in figure S4. The output of a fiber coupled LED (either 455 nm/ 530nm/ 625 nm) is coupled to a fiber (single mode), whose output is placed at the focal plane of an achromatic doublet lens for collimation. The collimated beam is transmitted through the meta-optic, whose point spread function is measured using a microscope setup consisting of a 50x objective, a tube lens and a CMOS sensor.

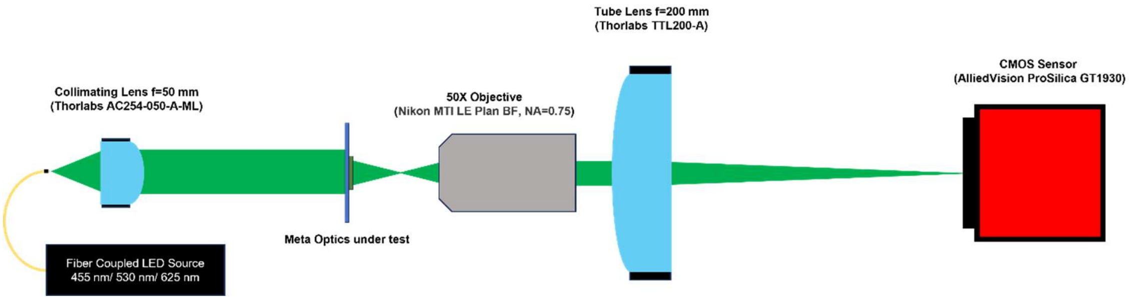

Figure S4. PSF measurement setup

S6. Captured Images and computational reconstruction.

The captured images of Figure 3 are presented in Figure S5

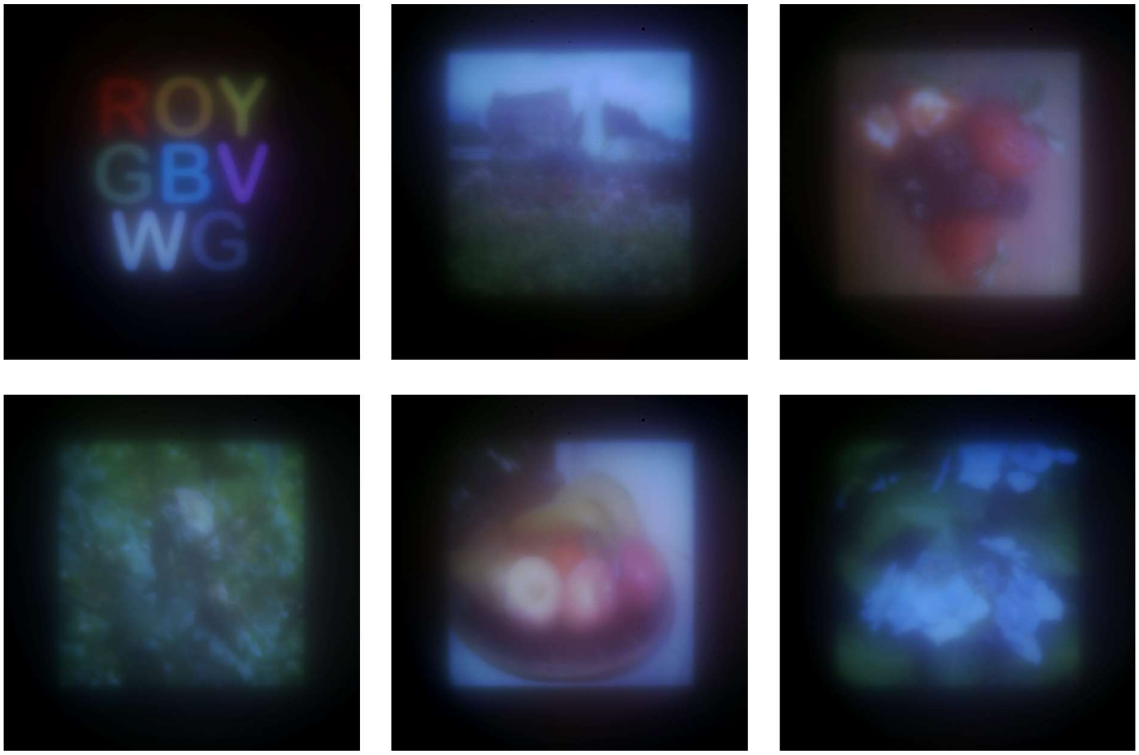

Figure S5. The as captured images before reconstruction, shown in Figure 4.

As discussed in the main text, we applied a computational backend to enhance the image quality. This approach was chosen similar to ref [30]. For completeness, we show examples of images before and after computational processing.

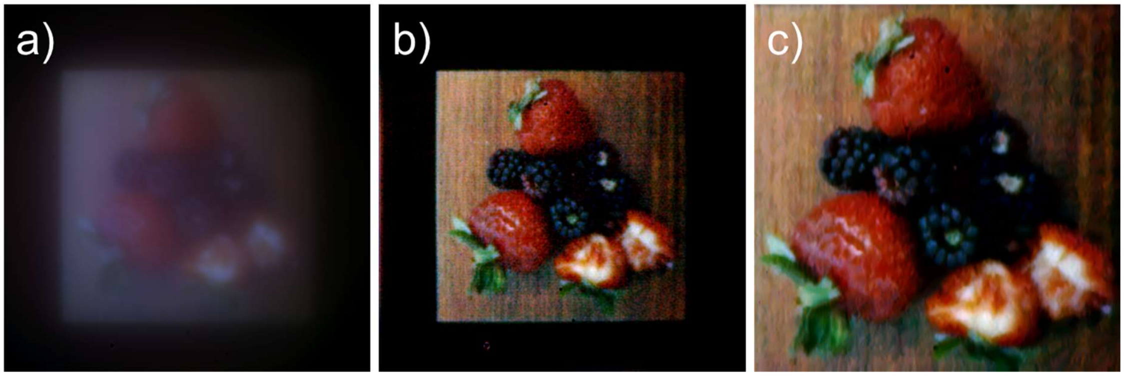

Figure S6. Example of an image during processing steps. a) Shows an image after capture. b) Shows the same image after Wiener deconvolution. c) Shows the image after denoising.

S7. Comparison with SiN meta-optics

We further compared with a SiN meta-optic with same parameter in simulation and experiment. As presented in Figure S6, we show the PSF for SoS (a,b) and SiN (c,d) meta-optics, which shows similar performance over the visible range. Moreover, we fabricated a SiN meta-optic with 1 mm aperture and performed imaging experiments in the same fashion with computational backend. The results are presented in Figure S6(e) for the SoS meta-optic and S6(f) for the SiN meta-optic. We essentially observe similar imaging performance after computation.

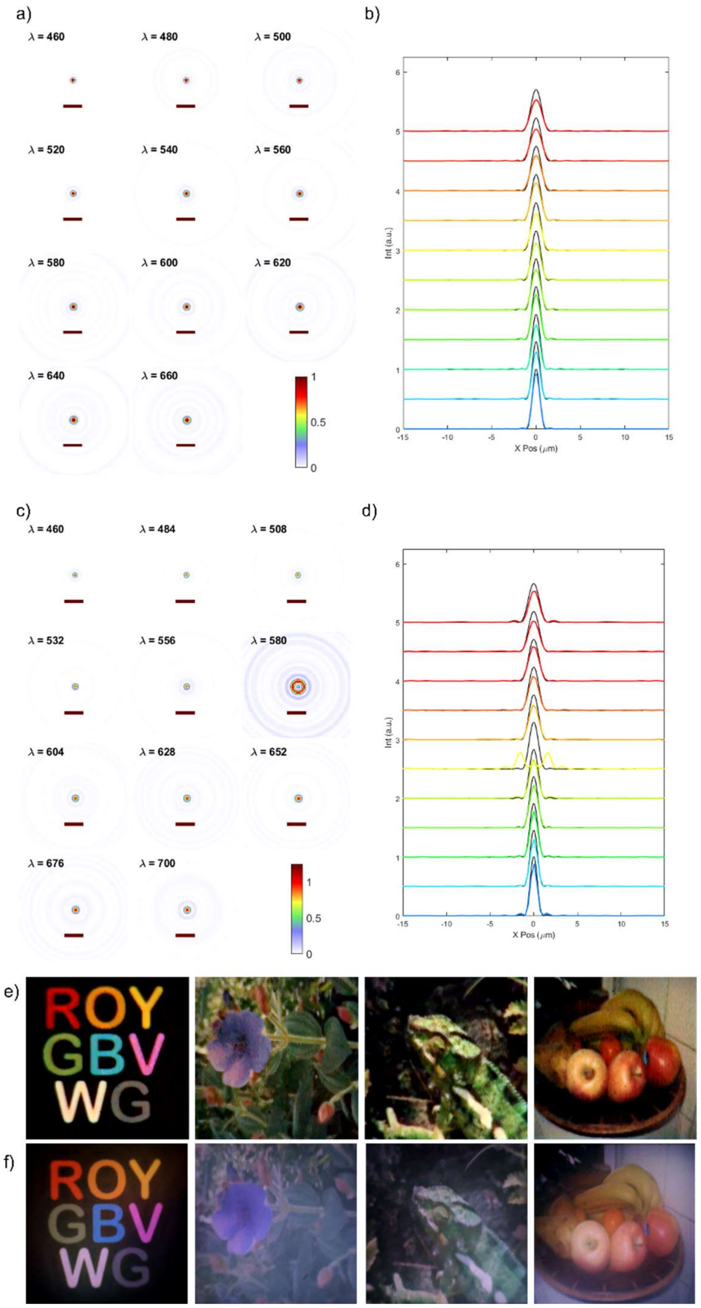

Figure S7. a) Simulated PSFs for SoS meta-optics at the specified wavelength throughout the visible range. b) Intensity Profile of the simulated PSF compared with a diffraction limited PSF in black for each wavelength. c) Simulated PSFs for SiN meta-optics at the specified wavelength throughout the visible range. d) Intensity Profile of the simulated PSF compared with a diffraction limited PSF in black for each wavelength. Images in (e) and (f) compare the on-par imaging performance of SoS meta-optic with SiN meta-optic.

References

[1] RefractiveIndex.INFO - Refractive index database, https://refractiveindex.info/, accessed: Nov., 2023.

[2] Y. Yang, G. Yoon, S. Park, S. D. Namgung, T. Badloe, K. T. Nam, J. Rho, *Advanced Materials* **2021**, *33*, 2005893.

[3] O. Goldberg, R. Gherabli, J. Engelberg, J. Nijem, N. Mazurski, U. Levy, *Advanced Optical Materials* **n.d.**, *n/a*, 2301612.

[4] D. Khmelevskaia, D. I. Markina, V. V. Fedorov, G. A. Ermolaev, A. V. Arsenin, V. S. Volkov, A. S. Goltaev, Yu. M. Zadiranov, I. A. Tzibizov, A. P. Pushkarev, A. K. Samusev, A. A. Shcherbakov, P. A. Belov, I. S. Mukhin, S. V. Makarov, *Applied Physics Letters* **2021**, *118*, 201101.

[5] Y. Wang, Z. Pan, Y. Yan, Y. Yang, W. Zhao, N. Ding, X. Tang, P. Wu, Q. Zhao, Y. Li, *Nanophotonics* **2024**, *13*, 3207.

[6] E. D. Palik, *Handbook of Optical Constants of Solids*, Academic Press, **1998**.

[7] D. E. Aspnes, A. A. Studna, *Phys. Rev. B* **1983**, *27*, 985.

[8] M. A. Green, *Solar Energy Materials and Solar Cells* **2008**, *92*, 1305.

[9] M. A. Green, M. J. Keevers, *Progress in Photovoltaics: Research and Applications* **1995**, *3*, 189.

[10] G. E. Jellison, *Optical Materials* **1992**, *1*, 41.

[11] C. Schinke, P. Christian Peest, J. Schmidt, R. Brendel, K. Bothe, M. R. Vogt, I. Kröger, S. Winter, A. Schirmacher, S. Lim, H. T. Nguyen, D. MacDonald, *AIP Advances* **2015**, *5*, 067168.

[12] G. Vuye, S. Fisson, V. Nguyen Van, Y. Wang, J. Rivory, F. Abelès, *Thin Solid Films* **1993**, *233*, 166.

[13] J. E. Fröch, L. Huang, Q. A. A. Tanguy, S. Colburn, A. Zhan, A. Ravagli, E. J. Seibel, K. F. Böhringer, A. Majumdar, *eLight* **2023**, *3*, 13.

[14] L. Huang, Z. Han, A. Wirth-Singh, V. Saragadam, S. Mukherjee, J. E. Fröch, Q. A. A. Tanguy, J. Rollag, R. Gibson, J. R. Hendrickson, P. W. C. Hon, O. Kigner, Z. Coppens, K. F. Böhringer, A. Veeraraghavan, A. Majumdar, **2023**, DOI: 10.48550/arXiv.2307.11385.

[15] W. T. Chen, A. Y. Zhu, V. Sanjeev, M. Khorasaninejad, Z. Shi, E. Lee, F. Capasso, *Nature Nanotech* **2018**, *13*, 220.

[16] S. Wang, P. C. Wu, V.-C. Su, Y.-C. Lai, M.-K. Chen, H. Y. Kuo, B. H. Chen, Y. H. Chen, T.-T. Huang, J.-H. Wang, R.-M. Lin, C.-H. Kuan, T. Li, Z. Wang, S. Zhu, D. P. Tsai, *Nature Nanotech* **2018**, *13*, 227.

[17] N. Mohammad, M. Meem, B. Shen, P. Wang, R. Menon, *Sci Rep* **2018**, *8*, 2799.

[18] W. T. Chen, A. Y. Zhu, J. Sisler, Z. Bharwani, F. Capasso, *Nat Commun* **2019**, *10*, 355.

[19] R. J. Lin, V.-C. Su, S. Wang, M. K. Chen, T. L. Chung, Y. H. Chen, H. Y. Kuo, J.-W. Chen, J. Chen, Y.-T. Huang, J.-H. Wang, C. H. Chu, P. C. Wu, T. Li, Z. Wang, S. Zhu, D. P. Tsai, *Nat. Nanotechnol.* **2019**, *14*, 227.

[20] M. Meem, S. Banerji, A. Majumder, C. Pies, T. Oberbiermann, B. Sensale-Rodriguez, R. Menon, *Applied Physics Letters* **2020**, *117*, 041101.

[21] F. Balli, M. A. Sultan, A. Ozdemir, J. T. Hastings, *Nanophotonics* **2021**, *10*, 1259.

[22] E. Tseng, S. Colburn, J. Whitehead, L. Huang, S.-H. Baek, A. Majumdar, F. Heide, *Nat Commun* **2021**, *12*, 6493.

[23] X. Xiao, Y. Zhao, X. Ye, C. Chen, X. Lu, Y. Rong, J. Deng, G. Li, S. Zhu, T. Li, *Light Sci Appl* **2022**, *11*, 323.

[24] Y. Hu, Y. Jiang, Y. Zhang, X. Yang, X. Ou, L. Li, X. Kong, X. Liu, C.-W. Qiu, H. Duan, *Nat Commun* **2023**, *14*, 6649.

[25] J. E. Fröch, P. K. Chakravarthula, J. Sun, E. Tseng, S. Colburn, A. Zhan, F. Miller, A. Wirth-Singh, Q. A. A. Tanguy, Z. Han, K. F. Böhringer, F. Heide, A. Majumdar, **2024**, DOI: 10.48550/arXiv.2402.06824.

[26] Z. Li, P. Lin, Y.-W. Huang, J.-S. Park, W. T. Chen, Z. Shi, C.-W. Qiu, J.-X. Cheng, F. Capasso, *Science Advances* **2021**, *7*, eabe4458.

[27] Z. Li, R. Pestourie, J.-S. Park, Y.-W. Huang, S. G. Johnson, F. Capasso, *Nat Commun* **2022**, *13*, 2409.

[28] A. V. Baranikov, E. Khaidarov, E. Lassalle, D. Eschimese, J. Yeo, N. D. Loh, R. Paniagua-Dominguez, A. I. Kuznetsov, *Laser & Photonics Reviews* **2024**, *18*, 2300553.

[29] M. Choi, J. Kim, S. Moon, K. Shin, S.-W. Nam, Y. Park, D. Kang, G. Jeon, K. Lee, D. H. Yoon, Y. Jeong, C.-K. Lee, J. Rho, *Nat. Mater.* **2025**, 1.

[30] L. Huang, J. Whitehead, S. Colburn, A. Majumdar, A. Majumdar, *Photon. Res., PRJ* **2020**, *8*, 1613.
